# Major hepatectomy with combined vascular resection for perihilar cholangiocarcinoma

**DOI:** 10.1093/bjsopen/zrab064

**Published:** 2021-08-05

**Authors:** T Sugiura, K Uesaka, Y Okamura, T Ito, Y Yamamoto, R Ashida, K Ohgi, S Otsuka, M Nakagawa, T Aramaki, K Asakura

**Affiliations:** 1 Division of Hepato-Biliary-Pancreatic Surgery; 2 Division of Plastic and Reconstructive Surgery; 3 Division of Diagnostic Radiology, Shizuoka Cancer Centre, Shizuoka, Japan

## Abstract

**Background:**

Hepatectomy with vascular resection (VR) for perihilar cholangiocarcinoma (PHCC) is a challenging procedure. However, only a few reports on this procedure have been published and its clinical significance has not been fully evaluated.

**Methods:**

Patients undergoing surgical resection for PHCC from 2002–2017 were studied. The surgical outcomes of VR and non-VR groups were compared.

**Results:**

Some 238 patients were included. VR was performed in 85 patients. The resected vessels were hepatic artery alone (31 patients), portal vein alone (37 patients) or both (17 patients). The morbidity rates were almost the same in the VR (49.4 per cent) and non-VR (43.8 per cent) groups (*P *=* *0.404). The mortality rates of VR (3.5 per cent) and non-VR (3.3 per cent) were also comparable (*P *>* *0.999). The median survival time (MST) was 45 months in the non-VR group and 36 months in VR group (*P *=* *0.124). Among patients in whom tumour involvement was suspected on preoperative imaging and whose carbohydrate antigen 19-9 (CA19-9) value was 37* *U/ml or less, MST in the VR group was significantly longer than that in the non-VR group (50 *versus* 34* *months, *P *=* *0.017). In contrast, when the CA19-9 value was greater than 37* *U/ml, MST of the VR and non-VR groups was comparable (28 *versus* 29* *months, *P *=* *0.520).

**Conclusion:**

Hepatectomy with VR for PHCC can be performed in a highly specialized hepatobiliary centre with equivalent short- and long-term outcomes to hepatectomy without VR.

## Teaser

Major hepatectomy with vascular (hepatic artery and/or portal vein) resection and reconstruction was performed in 85 consecutive patients with advanced perihilar cholangiocarcinoma. This surgery is technically demanding but can be performed with equivalent short- and long-term outcomes in a highly specialized hepatobiliary centre.

## Introduction

Perihilar cholangiocarcinoma (PHCC) is a highly intractable malignancy. One of the reasons for the intractability is that most patients are diagnosed with advanced disease at presentation. Thus, surgery for PHCC is one of the most difficult operations performed by hepatobiliary surgeons. Microscopic curative (R0) resection of the primary tumour is considered to be the only potentially curative treatment^1^. Because the anatomy of the hepatic hilum, in which the hilar bile duct is very close to the hepatic artery (HA) and portal vein (PV), is quite complicated, concomitant vascular resection (VR) is often required when R0 resection is performed. Advances in vascular anastomosis and reconstruction techniques based on transplant surgery have made combined vascular resection in hepatobiliary surgery possible and have contributed to the expansion of the surgical indications. Although some authors have revealed negative results of PV resection (PVR) for PHCC^2,^^3^, it has been recognized as an effective procedure for obtaining long-term survival^4–6^. The clinical significance of hepatectomy with HA resection (HAR) is still controversial^7–9^. Some meta-analyses took a cautious attitude towards VR for PHCC^10^^,^^11^. Recently, the Nagoya University group reported their experience in VR for PHCC in a large study population, and noted that it provided acceptable surgical outcomes and a substantial survival benefit^12^. However, it is the only institute in the world that specializes in surgery for PHCC. Thus, on the whole, the clinical benefits of VR – in terms of the surgical and oncological aspects – remain unclear.

Since its foundation in 2002, the authors have pursued radical care for patients with PHCC by performing aggressive surgical resection with VR. The present study was performed with the aim of reviewing the policy of aggressive surgical strategy for PHCC involving the HA and/or PV and clarifying the validity of VR.

## Methods

This study was approved by the institutional review board of Shizuoka Cancer Centre (J2020-17–2020-1–3).

A prospectively maintained hepatobiliary database was reviewed to identify patients who underwent resection of PHCC with curative intent between 2002 and 2017. In all patients, the diagnoses were confirmed histologically. The following data were retrieved for all patients included in the present analysis: sex, age, laboratory examination results, preoperative imaging diagnosis, perioperative data (blood loss, duration of operation, surgical procedure, morbidity and mortality), pathological findings and survival.

Perioperative outcomes were compared between patients with and without VR. A subgroup analysis was conducted to compare the overall survival between the patients with suspected vascular involvement who underwent VR and those who did not undergo VR.

### Preoperative assessment and management

The preoperative clinical evaluation involved laboratory and imaging studies, including multidetector-row computed tomography (MDCT)^13^^,^^14^, ultrasonography and cholangiography using either percutaneous transhepatic or endoscopic retrograde approach, and measurement of carbohydrate antigen 19-9 (CA19-9). Other imaging approaches, such as magnetic resonance imaging (MRI) and positron emission tomography, were utilized when needed. All serum CA19-9 values were measured after relief of jaundice. Preoperative volumetric assessment of the liver was performed using computed tomography. The functional reserve of the remnant liver was assessed based on the estimated indocyanine green (ICG) clearance from the future liver remnant^15^^,^^16^. An ICG clearance of the future liver remnant of 0.05 was adopted as the cut-off value for deciding liver resection^15^. Preoperative PV embolization (PVE) was carried out when right hepatectomy and left or right trisectionectomy were intended. This radiological intervention was performed via a percutaneous transhepatic approach approximately 2–3 weeks before hepatectomy. In cases of PVE, the liver function was assessed before and after PVE.

### Imaging analyses

**Fig. 1:**
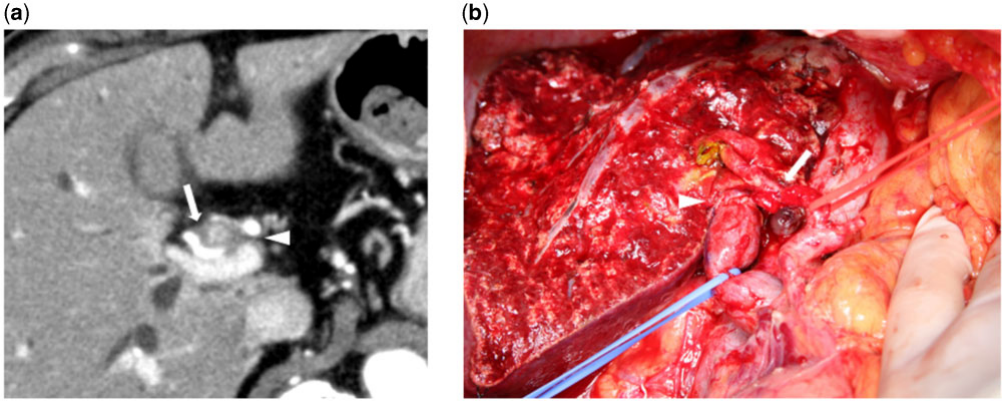
Perihilar cholangiocarcinoma **a** Computed tomography (axial view). The perihilar tumour is in contact with the hepatic artery (arrow) and portal vein (arrow head). **b** Image of left trisectionectomy, caudate lobectomy, with hepatic artery and portal vein resection and reconstruction. The right hepatic artery (arrow) and portal vein (arrow head) were reconstructed with end-to-end anastomosis

MDCT images were reviewed by experienced radiologists (mainly T.A. and K.A.) who were blinded to the other radiological data. The results were determined based on a consensus between at least two radiologists. Tumour contact with the HA and/or PV of the expected remnant liver side was defined as the absence of a visible fat layer between the tumour and HA and/or PV (*Fig. 1a*). Fat planes were assessed in two projections, including axial and coronal or sagittal projections, to examine the anteroposterior and craniocaudal relationships respectively. Finally, preoperative tumour staging, including tumour contact with the HA and/or PV, and the surgical strategy (type of hepatectomy and necessity of VR) were confirmed at the cancer board of the institute.

### Surgery

The contraindications for surgery were presence of distant metastasis, tumour involvement of the common HA, occlusion of the PV with cavernous transformation, involvement of peripheral HA and/or PV which were considered impossible to reconstruct, and evidence of macroscopic (bulky) para-aortic lymph node metastasis. Patients with proven para-aortic lymph node metastasis or positive peritoneal cytology by intraoperative examination were able to undergo surgery at the surgeon’s discretion. No patient underwent preoperative chemotherapy. The extent of hepatectomy was determined according to the preoperative diagnosis. All patients underwent regional lymph node dissection. If the tumour extended to the lower bile duct in the pancreatic head, pancreatoduodenectomy was performed simultaneously. In Bismuth type I/II PHCC, left hepatectomy with HAR was attempted instead of right hepatectomy, if the left liver functional reserve was insufficient^17^. An intraoperative frozen section diagnosis was performed for the proximal or distal bile duct margins. If the bile duct margins were positive, additional resection was performed as far as surgically possible.

Principally, concomitant resection of the HA or PV was carried out in cases in which macroscopic tumour involvement of these vessels was observed intraoperatively. Even if invasion was suspected based on the preoperative MDCT imaging, a vessel that could be freed from the tumour without difficulty was not resected. In contrast, even if preoperative images suggested no vascular invasion, resection was performed when the vessel could not be detached from the tumour. Prophylactic PVR using the no-touch technique^18^ was not applied. PV reconstruction was conducted by a hepatobiliary surgeon with continuous sutures using 5/0 or 6/0 monofilament suture thread. HA reconstruction was performed by plastic surgeons with an interrupted suture using 8/0 or 9/0 nylon under a surgical microscope^17^. All anastomoses were carried out in an end-to-end fashion (*Fig. 1b*). Anticoagulation therapy was not usually given during the postoperative period. In some cases with stenosis or deformity of the PV anastomosis, low-molecular-weight heparin was administered.

### Postoperative complications and follow-up

All postoperative complications and mortality after surgery were recorded and classified according to the Dindo–Clavien classification^19^. Postoperative liver failure and bile leakage were graded according to the International Study Group of Liver Surgery definition^20^^,^^21^.

Postoperative adjuvant chemotherapy was not routinely performed with the exception of cases that were included in a clinical trial^22^. Within the first 3 years after resection, follow-up examinations, including physical examinations, laboratory tests, assessment of tumour markers and computed tomography, were performed at 3-month intervals. If the patients had no signs of recurrence for 3 years after resection, follow-up examinations were performed at 6-month intervals. The median follow-up period of the censored patients was 73 months.

The site of recurrence was confirmed based on radiological or biopsy-proven evidence. Locoregional recurrence was specifically defined as a local ill defined mass along the hepatoduodenal ligament accompanied by positive findings of positron-emission tomography, increases in tumour markers and increases in size over time on serial imaging to detect disease progression.

### Statistical analyses

All statistical analyses were performed using SPSS^®^ version 25.0 (IBM, Armonk, New York, USA). Continuous variables were expressed as the median and range, and were dichotomized by referring to the minimum *P* values for survival analyses^23^. The Kaplan–Meier method was used to calculate the overall survival rates. These survival values were compared by a log rank test. The χ^2^ test or Fisher’s exact test were used to analyse categorical variables, as appropriate. The Mann–Whitney *U* test was used to analyse continuous variables. Univariable analyses were performed to determine the variables that were associated with survival. Variables with *P *<* *0.100 in a univariable analysis were included in a multivariable Cox proportional hazards regression analysis. Two-tailed *P *<* *0.050 was considered to indicate statistical significance.

## Results

**Fig. 2:**
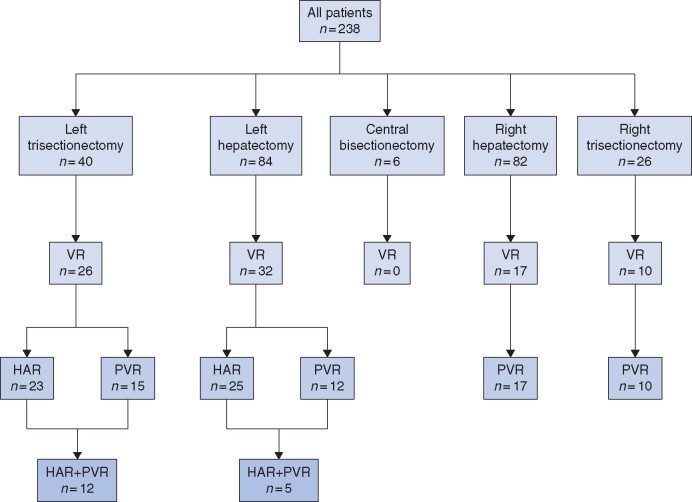
Diagram of vascular resection status and type of hepatectomy VR, vascular resection; HAR, hepatic artery resection; PVR, portal vein resection

A total of 238 patients underwent surgical resection for PHCC. During the same period, 36 patients with localized PHCC (no distant metastasis) did not undergo surgery for several reasons (locally advanced disease in 26 patients, poor general condition in 5, poor liver function in 4 and refusal of surgery in 1) and were instead treated with chemotherapy in the authors’ hospital (*TableS1,* supporting information**)**. Among patients who underwent surgery, VRs were performed in 85 (35.7 per cent) patients: HAR alone (31 patients, 13.0 per cent), PVR alone (37 patients, 15.6 per cent) and both HAR and PVR (17 patients, 7.1 per cent). At the preoperative evaluation by MDCT, 124 patients were diagnosed as having tumour contact with the HA and/or PV and VR was scheduled. Among them, 78 (62.9 per cent) patients underwent VR as planned. On the other hand, seven patients for whom VR was not planned underwent VR due to the intraoperative finding of macroscopic vascular invasion. The relationship between the type of hepatectomy and the type of VR is presented in *Fig. 2*. HAR was only performed in left-sided hepatectomy. The proportion of HAR in left trisectionectomy was approximately twice that in left hepatectomy. PVR was performed equally between right-sided and left-sided hepatectomy. Similarly to HAR, the proportion of PVR in right and left trisectionectomy was approximately twice that in right and left hepatectomy.

Several types of HA and PV reconstruction were carried out (*Table 1*). All HA reconstruction was performed with direct end-to-end anastomosis. No graft interposition was conducted. Seven types of artery were used for the proximal side and seven types of artery were selected for the distal side. In two patients, dual arterial anastomosis was performed. PVR included 53 segmental resections and one wedge resection. Two types of PV were used for the proximal side and four types were used for the distal side. In two patients, graft interposition using the left renal vein was required^24^.

**Table 1:** Type of arterial and portal vein anastomosis

	Proximal	Distal	Procedure (*n*)	Total no.
**Hepatic artery (*n* = 48)**	RHA	RHA	LH (6), LT (8)	14
	RHA	RPHA	LT (5)	5
	PHA	RHA	LH (10), LT (2)	12
	PHA	RPHA	LT (1)	1
	LHA	RHA	LH (2), LT (1)	3
	LHA	RPHA	LT (1)	1
	GDA	RHA	LH (5)	5
	GDA	RPHA	LT (1)	1
	RGA	RHA	LH (1)	1
	RGEA	RPHA	LT (2)	2
	A3	RPHA	LT (1)	1
	RHA	RAHA	LH (1)	1
	LHA	RPHA		
	GDA	A6	LT (1)	1
	LHA	A7		
**Portal vein (*n* = 54)**	MPV	LPV	RH (15), RT (10)	25
	MPV	RPV	LH (10), LT (7)	17 (1*)
	MPV	RPPV	LT (6)	6 (1^*^)
	MPV	MPV	LH (1), RH (2)	3
	RPV	RPV	LH (1)	1
	RPV	RPPV	LT (1)	1
	Wedge		LT (1)	1

*Graft anastomosis. RHA, right hepatic artery; PHA, proper hepatic artery; LHA, left hepatic artery; RPHA, right posterior hepatic artery; GDA, gastroduodenal artery; RGA, right gastric artery; RGEA, right gastroepiploic artery; A3, Couinaud's segment 3 branch; A6, Couinaud's segment 6 branch; A7, Couinaud's segment 7 branch; MPV, main portal vein; RPV, right portal vein; LPV, left portal vein; RPPV, right posterior portal vein; LH, left hepatectomy; LT, left trisectionectomy; RH, right hepatectomy; RT, right trisectionectomy.

The perioperative data are summarized in *Table 2.* In patients with VR, surgery was more invasive, the operation time was longer and the blood loss was greater. Several types of postoperative complications (Dindo–Clavien grade 3 or above) occurred in 109 (45.8 per cent) of the 238 patients; however, the incidence was almost identical between the VR and non-VR groups. Eight patients (3.4 per cent) died of postoperative complications: liver or multiple organ failure (5 patients), intra-abdominal abscess/sepsis (1 patient), aspiration pneumonia (1 patient) and intra-abdominal haemorrhage due to pseudoaneurysm of the gastroduodenal artery (1 patient). The latter patient who developed haemorrhage underwent hepatopancreatoduodenectomy without VR. The duration of postoperative hospital stay was comparable between the VR and non-VR groups. The perioperative data in patients with HAR (including simultaneous PVR) and PVR alone are presented in *TableS2* (supporting information). These results were almost comparable to the results of the overall VR cases. There was no particular increase in complications in the HAR group. When stratified by VR status, the morbidity and mortality rates were 52 per cent (16 patients) and 0 per cent (0 patients) respectively in HAR alone; 51 per cent (19 patients) and 5 per cent (2 patients) in PVR alone; and 42 per cent (7 patients) and 6 per cent (1 patient) in both HAR and PVR.

**Table 2:** Clinical, surgical and pathological outcomes according to VR status

	Non-VR (*n* = 153)	VR (*n* = 85)	***P*** §
Age (years)*	71 (37–87)	69 (40–84)	0.079
**Male**	101 (66.0)	60 (70.6)	0.469
CA19-9 (U/ml)*	55.0 (2–7288)	64.5 (2–5756)	0.199
**Bismuth type**			0.020
I	18	6	
II	19	15	
IIIa	38	11	
IIIb	39	17	
IV	39	36	
**Adjuvant chemotherapy**	8 (5.2)	6 (7.1)	0.565
**Adjuvant chemoradiotherapy**	8 (5.2)	5 (5.9)	0.832
**Pancreatoduodenectomy**	35 (22.9)	19 (22.4)	0.771
Time (min)*	543 (235–1171)	624 (390–984)	<0.001¶
Blood loss (ml)*	1329 (354–6692)	1824 (557–12 671)	<0.001¶
**Blood transfusion**	46 (30.1)	39 (45.9)	0.015
**Morbidity (C-D ≥grade 3)**	67 (43.8)	42 (49.4)	0.404
Incisional SSI	5 (3.3)	5 (5.9)	0.335
Organ/space SSI	28 (18.3)	15 (17.6)	0.901
Bile leakage	25 (16.3)	16 (18.8)	0.627
Pancreatic fistula	33 (21.6)	18 (21.2)	0.944
Liver failure	7 (4.6)	4 (4.7)	>0.999
Refractory ascites	6 (3.9)	6 (7.1)	0.289
Arterial thrombus	0 (0)	0 (0)	1
Portal vein thrombus	6 (3.9)	4 (4.7)	0.748
Liver abscess	2 (1.3)	2 (2.4)	0.618
Liver infarction	3 (2.0)	0 (0)	0.555
Intra-abdominal bleeding	5 (3.3)	1 (1.2)	0.425
Re-laparotomy	5 (3.3)	5 (5.9)	0.335
**Mortality**	5 (3.3)	3 (3.5)	>0.999
Hospital stay (days)*	24 (9–264)	24 (12–260)	0.938¶
**Histological grade (G2/G3)^†^**	82 (53.6)	55 (64.7)	0.097
**T status (pT3-4)^†^**	63 (41.2)	64 (75.3)	<0.001
**N status (pN1/2)^†^**	55 (35.9)	39 (45.9)	0.133
**M status (pM1)^†^**	8 (5.2)	7 (8.2)	0.361
**Perineural invasion**	115 (75.2)	78 (91.8)	0.002
**Liver invasion**	90 (58.8)	65 (76.5)	0.006
**Proximal ductal margin positive**	3 (2.0)	5 (5.9)	0.138
**Distal ductal margin positive**	3 (2.0)	0 (0)	0.555
**Dissection margin positive**	14 (9.2)	7 (8.2)	0.812
**R1 resection^†^**	19 (12.4)	12 (14.1)	0.709
**Microscopic invasion of resected hepatic artery^‡^**	–	30 (62.5)	–
**Microscopic invasion of resected portal vein^‡^**	–	38 (70.4)	–

Values in parentheses are percentages unless indicated otherwise;

*median (range). ^†^Union for International Cancer Control (8th edition) classification;

‡ 48 arterial resection cases and 54 portal vein resection cases were analysed; § χ2 test, except; ¶ Mann–Whitney U test. VR, vascular resection; CA19-9, carbohydrate antigen 19-9; D-C, Dindo–Clavien classification; SSI, surgical site infection.

The histopathological findings demonstrated that VR was significantly correlated with advanced pT stage and higher proportions of perineural invasion and liver invasion. However, the surgical margin status, including the proximal ductal margin, distal ductal margin and dissection margin, were equivalent. The proportions of microscopic invasion of the resected vessels were 62.5 per cent in HA and 70.4 per cent in PVR.

**Fig. 3:**
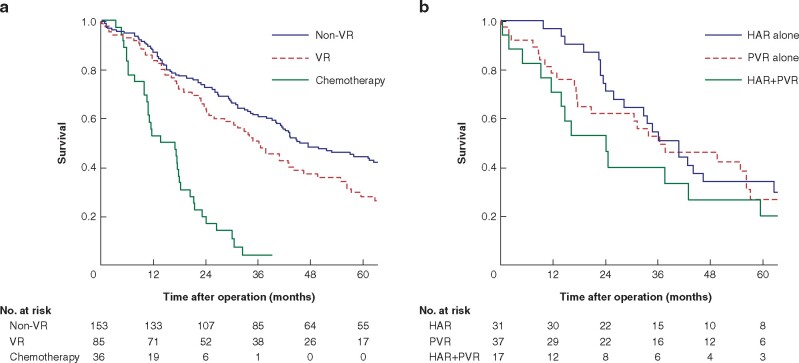
Kaplan–Meier curves showing survival **a** Survival of patients who underwent hepatectomy with and without vascular resection and who were treated with chemotherapy. *P* = 0.124 (non-VR *versus* VR), *P* < 0.001 (VR *versus* chemotherapy), *P* < 0.001 (non-VR *versus* chemotherapy). **b** Survival of patients according to vascular resection status. *P* = 0.894 (HAR alone *versus* PVR alone), *P* = 0.285 (HAR alone *versus* HAR+PVR), *P* = 0.407 (PVR alone *versus* HAR+PVR) (log-rank test). VR, vascular resection; HAR, hepatic artery resection; PVR, portal vein resection

The survival of the patients in the VR group (3- and 5-year survival rates and median survival time (MST): 49.2 per cent, 27.7 per cent and 36 months) tended to be worse in comparison with the non-VR group (60.5 per cent, 44.2 per cent and 45 months); however, the difference was not statistically significant (*P *=* *0.124) (*Fig. 3a*). This was significantly better than that of patients who did not undergo surgery but were treated with chemotherapy (3.5 per cent, 0 per cent and 14 months) (*P *<* *0.001). Among the 85 patients in the VR group, 17 (20.0 per cent) survived for more than 5 years (14 patients without recurrence). Among the patients in the VR group, the survival of patients who received both HAR and PVR tended to be worse than that of patients treated with other procedures; however, none of the differences were statistically significant: 3- and 5-year survival rates and MST were 51 per cent, 34 per cent and 40 months in HAR alone, 52 per cent, 27 per cent and 38 months in PVR alone and 40 per cent, 20 per cent and 24 months in both HAR and PVR (*Fig. 3b*).

*Table 3* shows the results of the univariable and multivariable analyses of prognostic factors in patients with PHCC (excluding 8 patients who died due to postoperative complications). A preoperative CA19-9 value of greater than 37 U/ml, histological grade G2/G3, and the presence of lymph node metastasis, positive R status and positive M status were identified as significant prognostic factors. VR was not a significant prognostic factor.

**Table 3:** Prognostic factors for perihilar cholangiocarcinoma^*^

		Univariable analysis		Multivariable analysis	
	*n*	**MST (months)**	*P*	Hazard ratio	***P* ^‡^**
**CA19-9 (U/ml)**					
≤37	97	79	<0.001	1	
>37	133	36		1.78 (1.25–2.50)	0.001
**Vascular resection**					
No	148	52	0.092	1	
Yes	82	36		1.03 (0.72–1.47)	0.890
**Histological grade^†^**					
G1	99	61	<0.001	1	
G2/G3	131	36		1.64 (1.16–2.33)	0.006
**T status^†^**					
pT1–2	108	57	0.004	1	
pT3–4	122	38		1.24 (0.87–1.77)	0.228
**N status^†^**					
pN0	140	68	<0.001	1	
pN1/2	90	31		1.64 (1.22–2.34)	0.007
**M status^†^**					
pM0	217	46	0.001	1	
pM1	13	31		2.10 (1.10–4.01)	0.025
**Lymphovascular invasion**					
No	62	73	0.002	1	
Yes	168	39		1.26 (0.80–1.99)	0.325
**Perineural invasion**					
No	44	112	0.003	1	
Yes	186	41		1.20 (0.71–2.05)	0.500
**Portal vein invasion**					
No	170	46	0.103		
Yes	60	36			
**Hepatic artery invasion**					
No	178	45	0.317		
Yes	52	38			
**R status**					
R0	202	47	<0.001	1	
R1	28	29		1.66 (1.05–2.62)	0.030

*Analyses with 230 patients (excluding 8 deceased patients). Values in parentheses are 95 percent confidence intervals.

† Union for International Cancer Control (8th edition) classification. ^‡^ Cox proportional hazards regression analysis. CA19-9, carbohydrate antigen 19-9; MST, median survival time.

**Fig. 4:**
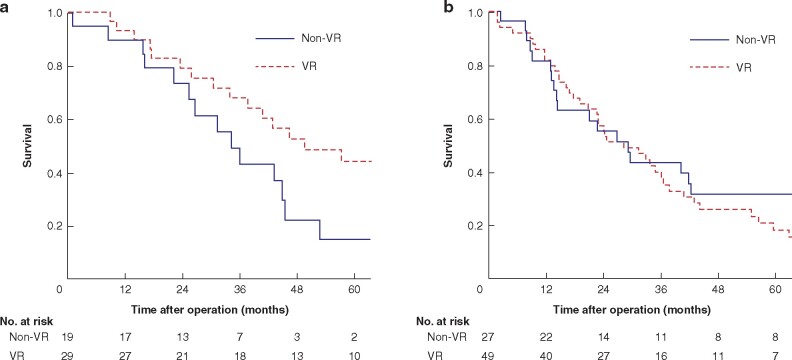
Survival of patients with suspicious vascular invasion findings on preoperative multidetector-row computed tomography imaging **a** In the group with CA19-9 37 U/ml or less, *P* = 0.017. **b** In the group with CA19-9 greater than 37 U/ml, *P* = 0.520 (log-rank test). VR, vascular resection

For the validation of the authors’ strategy of VR, a subgroup analysis of 124 patients with suspected vascular invasion was conducted (tumour contact with HA and/or PV on MDCT imaging). The CA19-9 value was used for stratification of patients, as it was the only prognostic factor that could be obtained preoperatively. The survival of patients with suspicious vascular invasion findings are presented in *Fig. 4*. Among patients with CA19-9 of 37 U/ml or less, the survival of the VR group (3- and 5-year survival rate and MST: 67.7 per cent, 44.0 per cent and 50 months) was significantly better than that of the non-VR group (42.8 per cent, 14.7 per cent and 34 months) (*P *=* *0.017). In contrast, among patients with CA19-9 greater than 37 U/ml, the survival of the patients was comparable, irrespective of the presence or absence of VR: 3- and 5-year survival rates and MST were 43.3 per cent, 31.5 per cent and 28 months in the VR group and 37.3 per cent, 18.0 per cent and 29 months in the non-VR group (*P *=* *0.520). The relationships between the dissected margin status/locoregional recurrence and VR status are presented in *Table 4*. The proportions of positive dissected margin and locoregional recurrence in all patients were identical between the VR and non-VR groups. Among patients with findings suggestive of vascular invasion, these proportions in the non-VR group were approximately twice those in the VR group, irrespective of CA19-9 value; however, the difference was not statistically significant.

**Table 4:** The relationship between the dissected margin status/locoregional recurrence and VR status

	non-VR	VR	***P* ^†^**
**All patients**	*n* = 153	*n* = 85	
Positive dissected margin	14 (9.1)	7 (8.2)	0.812
Local recurrence	27 (17.6)	15 (17.6)	1.000
Patients with suspicious findings of vascular invasion* and CA19-9 ≤37 **U/ml**	*n* = 19	*n* = 29	
Positive dissected margin	5 (26.3)	3 (10.3)	0.236^‡^
Local recurrence	7 (36.8)	5 (17.2)	0.125
Patients with suspicious findings of vascular invasion* and CA19-9 >37 **U/ml**	*n* = 27	*n* = 49	
Positive dissected margin	5 (18.5)	4 (8.2)	0.266^‡^
Local recurrence	9 (33.3)	10 (20.4)	0.271

Values in parentheses are percentages;

*preoperative multidetector-row computed tomography imaging; ^†^χ2 test, except; ^‡^ Fisher’s exact test. VR, vascular resection; CA19-9, carbohydrate antigen 19-9.

## Discussion

Vascular invasion remains a major obstacle in the treatment of PHCC. The hilar cholangiocarcinoma expert consensus statement presented criteria for defining unresectable cases: unilateral segmental extension (atrophy) with contralateral vascular inflow^25^. In the present study, VR was performed aggressively for the treatment of advanced PHCC in patients with a higher T stage and higher proportions of perineural and liver invasion. HAR and PVR were relatively safe in the perioperative period and offered acceptable long-term survival. This result is consistent with the findings reported by Mizuno and colleagues^12^. However, because this procedure is technically demanding, it should be interpreted as a result at a high-volume hepatobiliary centre. Hepatectomy with and without bile duct resection is highly invasive and the volume–outcome relationship has been proven^26^. Therefore, centralization of the most extensive resections in high-volume centres may have played a role.

There have been no randomized studies of PVR for PHCC and no such studies are expected to be planned in the future. Thus, the effectiveness of PVR can only be inferred from previous studies. In general, PVR is considered to contribute to a favourable prognosis^2^^,^^4^^,^^5^^,^^7^^,^^18^^,^^27^. Therefore, in the leading hepatobiliary centres around the world, PVR has been performed aggressively and has now become a routine procedure^4^^,^^8^^,^^27–31^. Two recent meta-analyses concluded that PVR in patients with gross involvement of the PV is feasible in terms of the short- and long-term results^32^^,^^33^. Wu and co-workers^32^ reported that a subgroup analysis demonstrated that, in centres with more experience or studies published after 2007, combined PVR was not associated with significantly higher postoperative morbidity or mortality. The Japanese guidelines for biliary tract cancers note that PVR is useful for patients with PV invasion because the prognosis of patients treated with PVR is significantly better than that of unresected patients^34^.

Regarding HAR, the benefits are controversial. Some studies have reported that HAR can be performed safely and contribute to a better prognosis^8^^,^^35^^,^^36^, but some studies have reported that HAR is associated with high rates of morbidity and mortality, without a survival benefit^7^. Noji and colleagues^37^ reported their experience, and after propensity score matching, no significant difference in postoperative morbidity or survival was observed between patients who underwent HAR and those who did not. The present study revealed that the survival of patients who received HAR alone was similar to that of patients who received PVR alone. Thus, HAR alone is considered to be feasible.

HA reconstruction is more complicated than PV reconstruction. The HA shows various anatomical variations^38^. A full understanding of these variations is crucial for successful surgical resection of PHCC. MDCT images and its three-dimensional reconstruction images allow recognition of the detailed branching pattern and running course of the HA without angiography^39^^,^^40^. MDCT is also useful for evaluating the range of tumour infiltration^14^. In addition to anatomical recognition, cooperation with vascular or plastic surgeons is another key to the success of HA reconstruction. Nagino and colleagues^8^ emphasized that the excellent technique of vascular and plastic surgeons was largely responsible for their favourable results. To perform this challenging operation successfully, a multidisciplinary team approach with hepatobiliary surgeons, vascular/plastic surgeons and radiologists is mandatory. Recently, Hu and co-workers reported that HAR without reconstruction is also a safe and feasible surgical procedure for selected cases: cases with severe infiltration of the HAs combined with obviously decreased blood flow detected by intraoperative ultrasound, or those in which infiltration of the HAs is at a higher position in combination with a small lumen^41^. However, although there was no significant difference, the HAR without reconstruction group showed a slightly higher hepatic failure rate compared with the HAR with reconstruction group (13.8 *versus* 5.9 per cent).

The survival benefit of simultaneous HAR and PVR for PHCC is less clear because there have been few reports with a limited number of cases on this extended approach. In the study period, simultaneous HAR and PVR was performed in 17 patients. This procedure is supremely challenging. It is noteworthy that 70 per cent (12 of 17) of these procedures were performed with accompanying left trisectionectomy. Among the various types of hepatectomy, left trisectionectomy is the most technically demanding procedure^42–45^. A report using the annual safety reports provided by Japanese Society of Hepato-Biliary-Pancreatic Surgery board-certified training institutions reported that the 90-day mortality rate of patients who underwent left trisectionectomy was 10.3 per cent^46^. In that sense, the patients who required simultaneous HAR and PVR were in a difficult situation. Nagino and colleagues^8^ reported the highest volume of 50 consecutive cases of simultaneous HAR and PVR with acceptable rates of morbidity (54 per cent) and mortality (2 per cent). Although the number of cases in the present study was one-third the number in the study of Nagino and colleagues^8^, this procedure was performed with equivalent rates of morbidity and mortality. As for the long-term outcomes, the patients who underwent simultaneous HAR and PVR had relatively worse survival, with 3- and 5-year survival rates of 40 and 20 per cent. However, this was almost identical to the report by Nagino and colleagues^8^ (36.3 and 30.3 per cent). Considering these results, simultaneous HAR and PVR is not a contraindication; however, it should be performed carefully.

In patients in whom tumour contact with major vessels was suspected based on preoperative MDCT imaging and CA19-9 values of 37 U/ml or less, the VR group had significantly better survival than the non-VR group. One possible reason for this difference is the relatively higher incidence of positive dissected margin and local recurrence in the non-VR group. Although vessels could be freed from the tumour and the dissected margin was found to be negative, unidentified cancer exposure could be responsible for these results. In contrast, when the CA19-9 value was greater than 37 U/ml, survival in the VR and non-VR groups was comparable. Serum CA19-9 is reported to be increased in approximately 60–70 per cent of patients with cholangiocarcinoma, and the preoperative CA19-9 level has been shown to be inversely correlated with survival in patients with resectable disease^47^^,^^48^. Wang and co-workers^49^ reported that a higher preoperative CA19-9 value was associated with early recurrence after resection of PHCC. Coelho and colleagues^50^ described that a higher CA19-9 level was associated with distant metastasis. Based on these results, patients with a higher preoperative CA19-9 levels are more likely to have a higher tumour burden and systemic disease. In contrast, patients with CA19-9 values of 37 U/ml or less may have only local disease. In that sense, these patients were the most appropriate candidates for VR and it would be appropriate to abandon attempts to dissect the vessels from the tumour.

The present study was associated with several limitations, including its retrospective design and the fact that it was performed in a single centre without external validation. The number of subjects was not sufficient to draw broad interpretations. A large multicentre series would be necessary to draw definitive conclusions on the benefits of VR for PHCC.

## Acknowledgements 

This study received no funding.

*Disclosure*. The authors declare no conflict of interest.

## Supplementary material

Supplementary material is available at *BJS Open* online.

## Supplementary Material

zrab064_Supplementary_Data
