# Supplementary material for: Major hepatectomy with combined vascular resection for perihilar cholangiocarcinoma
Source: BJS Open. 2021 Aug 5;5(4):zrab064. doi: 10.1093/bjsopen/zrab064 (PMC8342931; doi:10.1093/bjsopen/zrab064)
Supplement: zrab064_Supplementary_Data [file zrab064_supplementary_data.docx]

| Supplementary Table 1. Details of patients undergoing chemotherapy only | | |
| --- | --- | --- |
| Age (years)* |  | 70 (31-80) |
| Sex |  |  |
| Male |  | 22 |
| Female |  | 14 |
| CA 19-9 (U/ml)* |  | 236 (2-32351) |
| Bismuth type |  |  |
| II |  | 4 |
| IIIa |  | 13 |
| IIIb |  | 7 |
| IV |  | 12 |
| T status† |  |  |
| cT3 |  | 4 |
| cT4 |  | 32 |
| N status† |  |  |
| cN0 |  | 15 |
| cN1 |  | 21 |
| M status† |  |  |
| cM0 |  | 36 |
| cM1 |  | 0 |
| *median (range); †UICC (8th) classification | | |

| Supplementary Table 2. The clinical, surgical, and pathological outcomes according to VR status | | | |
| --- | --- | --- | --- |
|  | HAR (n=48) | PVR (n=37) | *p* |
| Age (years)* | 71 (50-84) | 68 (40-81) | 0.588 |
| Sex (male) | 32 (66.7) | 28 (75.7) | 0.366 |
| CA 19-9 (U/ml)* | 107 (2-5756) | 39 (2-1130) | 0.043 |
| Bismuth type |  |  | 0.017 |
| I | 3 | 3 |  |
| II | 10 | 5 |  |
| IIIa | 1 | 10 |  |
| IIIb | 11 | 6 |  |
| IV | 23 | 13 |  |
| Adjuvant chemotherapy | 3 (6.3) | 3 (8.1) | 0.999 |
| Adjuvant chemoradiotherapy | 3 (6.3) | 2 (5.4) | 0.999 |
|  |  |  |  |
| Pancreatoduodenectomy | 10 (20.8) | 9 (24.3) | 0.702 |
| Time (min)* | 646 (399-971) | 566 (390-984) | <0.001 |
| Blood loss (ml)* | 1681 (799-4641) | 1652 (390-12671) | 0.989 |
| Blood transfusion | 23 (47.9) | 16 (43.2) | 0.668 |
| Morbidity (D-C >grade 3) | 23 (47.9) | 19 (51.4) | 0.754 |
| Incisional SSI | 2 (4.2) | 3 (8.1) | 0.649 |
| Organ/space SSI | 7 (14.6) | 8 (21.6) | 0.399 |
| Bile leakage | 7 (14.6) | 9 (24.3) | 0.255 |
| Pancreatic fistula | 11 (22.9) | 7 (18.9) | 0.655 |
| Liver failure | 1 (2.1) | 3 (8.1) | 0.313 |
| Refractory ascites | 3 (6.3) | 3 (8.1) | 0.999 |
| Arterial thrombus | 0 (0) | 0 (0) | 1 |
| Portal vein thrombus | 4 (8.3) | 0 (0) | 0.129 |
| Liver abscess | 1 (2.1) | 1 (2.7) | 0.999 |
| Liver infarction | 0 (0) | 0 (0) | 1 |
| Intra-abdominal bleeding | 0 (0) | 1 (2.7) | 0.435 |
| Relaparotomy | 2 (4.2) | 3 (8.1) | 0.649 |
| Mortality | 1 (2.1) | 2 (5.4) | 0.577 |
| Hospital stays (days)* | 23 (13-260) | 24 (12-82) | 0.540 |
|  |  |  |  |
| Histological grade (G2/G3)† | 30 (62.5) | 25 (67.6) | 0.628 |
| T status (pT3-4)† | 35 (72.9) | 29 (78.4) | 0.563 |
| N status (pN1/2)† | 21 (43.8) | 18 (48.6) | 0.653 |
| M status (pM1)  † | 1 (2.1) | 6 (16.2) | 0.040 |
| Perineural invasion (positive) | 45 (93.8) | 33 (89.2) | 0.448 |
| Liver invasion (positive) | 41 (85.4) | 24 (64.9) | 0.039 |
| Proximal ductal margin (positive) | 0 (0) | 5 (13.5) | 0.013 |
| Distal ductal margin (positive) | 0 (0) | 0 (0) | 1 |
| Dissection margin (positive) | 4 (8.3) | 3 (8.1) | 0.999 |
| R1 resection† | 4 (8.3) | 8 (21.6) | 0.081 |

Values in parentheses are percentages unless indicated otherwise; *median (range); †UICC (8th) classification; HAR, hepatic artery resection; PVR, portal vein resection; CA 19-9, carbohydrate antigen 19-9; D-C, Dindo-Clavien classification; SSI, surgical site infection
